# Effects of Motor Training on Accuracy and Precision of Jaw and Finger Movements

**DOI:** 10.1155/2019/9593464

**Published:** 2019-11-18

**Authors:** Yinan Chen, Song Wu, Zhengting Tang, Jinglu Zhang, Lin Wang, Linfeng Yu, Kelun Wang, Peter Svensson

**Affiliations:** ^1^Orofacial Pain & TMD Research Unit, Institute of Stomatology, Department of Polyclinic, Affiliated Hospital of Stomatology, Nanjing Medical University, Nanjing, China; ^2^Jiangsu Key Laboratory of Oral Diseases, Nanjing Medical University; Department of Polyclinic, Affiliated Hospital of Stomatology, Nanjing Medical University, Nanjing, China; ^3^Department of Dentistry, BenQ Medical Center, The Affiliated BenQ Hospital of Nanjing Medical University, Nanjing, Jiangsu Province, China; ^4^Center for Sensory-Motor Interaction (SMI), Aalborg University, Aalborg, Denmark; ^5^Section of Orofacial Pain and Jaw Function, Department of Dentistry and Oral Health, Aarhus University, Denmark; ^6^Department of Dental Medicine, Karolinska Institutet, Huddinge, Sweden; ^7^Scandinavian Center for Orofacial Neurosciences (SCON), Denmark

## Abstract

**Objective:**

To compare the effects of training of jaw and finger movements with and without visual feedback on precision and accuracy.

**Method:**

Twenty healthy participants (10 men and 10 women; mean age 24.6 ± 0.8 years) performed two tasks: a jaw open-close movement and a finger lifting task with and without visual feedback before and after 3-day training. Individually determined target positions for the jaw corresponded to 50% of the maximal jaw opening position, and a fixed target position of 20 mm was set for the finger. Movements were repeated 10 times each. The variability in the amplitude of the movements was expressed as percentage in relation to the target position (*D*_accu_—accuracy) and as coefficient of variation (CV_prec_—precision).

**Result:**

*D*_accu_ and CV_prec_ were significantly influenced by visual feedback (*P* = 0.001 and *P* < 0.001, respectively) and reduced after training jaw and finger movements (*P* < 0.001). *D*_accu_ (*P* = 0.004) and CV_prec_ (*P* = 0.019) were significantly different between jaw and finger movements. The relative changes in *D*_accu_ (*P* = 0.017) and CV_prec_ (*P* = 0.027) were different from pretraining to posttraining between jaw and finger movements.

**Conclusion:**

The accuracy and precision of standardized jaw and finger movements are dependent on visual feedback and appears to improve more by training in the trigeminal system possibly reflecting significant neuroplasticity in motor control mechanisms.

## 1. Introduction

Neuroplasticity is one of the most prominent features of the central nervous system, which plays a role in function to store information in memory associated with learning [1]. In healthy humans, gain in overall motor performance, reflective of motor learning, has been associated with neuroplasticity of the motor cortical territories corresponding to the trained muscles and can occur after short-term (hours to days) motor training regimes [2–4].

Previous studies have shown that the neuroplasticity of corticomotor pathways can be established after a series of repetitive motor tasks such as tooth clenching [5, 6], tongue movements, hand movements [7, 8], and leg movements [9]. It has been suggested that motor training results in improvements in the precision of the task performance with increased representation of the trained muscle in the motor cortex [3, 8].

Repetitive jaw open-close movements are one of the basic components of physiological jaw motor actions during mastication. Open-close jaw movements must achieve a predetermined requirement with high precision in mastication, a complex biomechanical process, involving positioning food between the teeth, holding, breaking it down into pieces to prepare it for swallowing [10]. The essential rhythm of jaw movements is determined by a group of neurons in the brain stem termed the masticatory CPG (central pattern generator) [10–12]. The CPG is initiated by the inputs from higher centers (motor cortex) and is influenced by somatosensory information from the orofacial mechanoreceptors [10, 11]. Therefore, it is suggested that the somatosensory cortex and the motor cortex are important in the mastication process, e.g., chewing and swallowing. The primary motor cortex is involved in the initiation, control, and execution of the jaw motor functions and also plays an important role in the acquisition of new motor skills [13].

Recent research on oral motor performance has demonstrated the ability to increase performance and skill acquisition during different orofacial motor tasks, for example, manipulation and splitting spherical pieces of candy into two almost equal halves following a short-term training (about 30 min) [14]. The results of that study showed that the participants were able to successfully perform the task with increased levels of precision following a short-term training. Another recent study by Kumar et al. investigated the dynamic changes in accuracy and precision during a simple oral and digital motor task involving a controlled and a ballistic force. That study demonstrated differences between accuracy and precision of training-related dynamic modulations of forces due to repeated performance; i.e., it may be important to consider both accuracy and precision in the evaluation of neuroplastic changes in motor skills [15].

There may also be differences in motor control programs between hand and jaw muscles reflecting anatomical and functional differences between the trigeminal and spinal system. Iida et al. used functional magnetic resonance imaging (fMRI) to provide more information on the central processing mechanisms underlying awake bruxism and directly compared cerebral activity between bilateral light fist clenching and light tooth clenching [16]. Interestingly, the results showed that a less complex pattern of cerebral activity was induced by the hand motor task compared to the performance of the tooth clenching [16]. Another recent study recorded electromyography (EMG) and force in repetitive finger pinch and tooth clenching tasks and demonstrated that motor learning for the jaw motor system is more likely to occur than that for the hand motor system [17]. It seems that information on neuroplastic changes evoked by motor training cannot directly be extrapolated from the spinal system to the trigeminal system.

In previous studies, the behavioral consequences of motor learning have been characterized by two descriptive statistics, a mean and a variance, the former meaning accuracy, also referred to as “constant error” and the latter meaning precision, sensitivity, discrimination threshold, or “temporal variance” [18, 19]. However, no study has so far compared both the accuracy and precision of jaw and finger movements in terms of training-induced neuroplastic effects and motor performance.

The hypothesis of the present study was that the accuracy and precision of jaw and finger movements would increase after short-term training paradigms with no differences between the trigeminally and spinally innervated muscles.

## 2. Materials and Methods

### 2.1. Participants

Twenty young adults (ten men and ten women; mean age 24.6 ± 0.8 years) were recruited among the students at Nanjing Medical University. All participants were healthy individuals with no signs or symptoms of pain in the head, face, and the right index finger and absence of any jaw movement restrictions that involved the masticatory musculature, the temporomandibular joint, and associated structures. The exclusion criteria included past trauma to the jaw and right arm or hand, a history of systemic illnesses to joints or related muscles, ongoing dental treatment, mental disorders, peripheral neuropathy, presence of any acute or chronic orofacial pain conditions or taking medications for pain, or neurological diseases. The study was conducted in accordance with the guidelines set forth in the Declaration of Helsinki II, and informed consent was obtained from all participants prior to participation. The study was approved by the Nanjing Medical University Research Ethics Committee (No. PJ2015-040-001).

### 2.2. Recording Equipment

**Figure 1:**
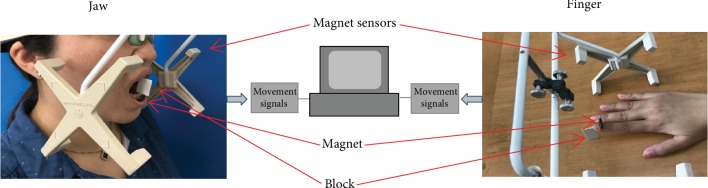
Illustration of the movement recording equipment applied to the jaw and finger. Magnet attached to lower incisors in the midline or to the nail of the right index finger. Magnet sensors (4 × 2) in the headset recorded the vertical dimension of the jaw and finger movements. Individual plastic blocks were fabricated to guide participants to the target positions.

A kinesiograph (K7/CMS; Myotronics, Inc., USA) was used to record jaw and finger movements with an accuracy of 0.1 mm and a sampling frequency of 50 Hz. An array attached to a headset containing 8 magnetic sensors tracked the motion of a magnet (CMS Magnet; Myotronics, Inc., USA) attached to the lower incisors in the midsagittal plan or the nail of the right index finger (Figure 1). The magnet was positioned based on anatomical guidelines and reference points with a test-retest variability < 1 mm. The movement data was sampled, stored, and analyzed by a commercial software program (K7 Program, Myotronics, Inc., USA).

### 2.3. Recording Procedure

**Figure 2:**
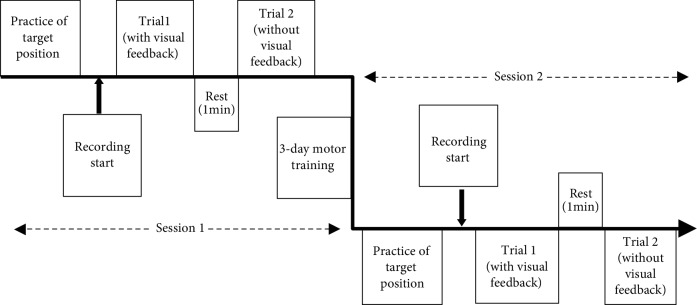
Schematic diagram of the experimental design: session 1—pretraining, session 2—post-training, trial 1—movements with visual feedback, and trial 2—movements without visual feedback.

All participants were examined twice in two separate sessions in this study (Figure 2). In each session, participants were asked to perform 10 repeated jaw movements and finger movements to a target position during two trials: trial 1 with visual feedback and trial 2 without visual feedback. In trial 1, all participants kept their eyes open to control their finger movements and they were given a mirror to control their jaw movements. In trial 2, the participants received no visual feedback from the jaw and finger movements as they were asked to keep their eyes closed. The 10 repeated open-close jaw movements and finger lifting movements were considered one trial, respectively. In session 1, all movements were without any prior motor training. In session 2, all participants had repeated the same series of movements for 3 days: three times of motor training for the jaw and finger each day. The pace of the movement was controlled by a metronome set to 10 beats/min (auditory cue) to maintain a similar speed in each trial. All recordings were performed by the same examiner.

### 2.4. Jaw Movements

The participants were seated in a chair in a comfortable upright position with a straight back. The jaw tracking device was mounted on the participants in accordance with previously published papers [20].

At the beginning of the test, participants were instructed to open their jaw as wide as possible without feeling pain starting from the intercuspal position with teeth in light contact for three times to obtain the mean value of the maximal jaw opening. A simple ruler was used to determine the interincisal distance and the horizontal overbite. Then the task was to perform 10 repeated open-close movements to an individually adjusted target position corresponding to 50% of the maximal jaw opening position adjusted for the individual differences in vertical overbite. A plastic block (block 1) was cut for each participant to reproduce the 50% of the maximal jaw opening position as the target position (Figure 1). Prior to each trial, the participants were instructed with the aid of block 1 to open their jaw to reach the target position. Then the open-close jaw movement was performed 10 times without the help of block 1.

### 2.5. Finger Movements

The participants were comfortably seated in a chair and placed their right hand and arm on the table with the palm of the hand downwards without pressure or efforts. The magnet (CMS Magnet; Myotronics, Inc., USA) was attached to the nail of the right index finger with the hand adjusted to the center of the headset of kinesiograph device (Figure 1). The participants were instructed to lift only the index finger from the resting position on the table in a vertical direction to an amplitude corresponding to 20 mm defined as the target position at the beginning of the test. Another plastic block (block 2) was cut to reproduce the target position for each participant. Prior to each trial, participants were instructed to lift the finger to the target position with the help of block 2 and then the finger movements were performed 10 times without the aid of block 2. We connected the magnet in a straight line and made a vertical line at the midpoint of the line. In the experiment, we placed the midpoint in the center of the nail and overlapped the long axis of the index finger and the vertical line completely in order to minimize variation and to standardize recording conditions.

An additional control experiment was performed in a separate session in eleven other healthy individuals (3 men and 8 women, mean age: 26.5 ± 1.9 years) to determine accuracy and precision at different ranges of amplitudes of the finger lifting task. The amplitude was set at 10, 20, 40, and 60 mm to cover the 50% of the maximal index finger lift capacity. A total of 10 repetitions at the same speed as described above and with the exact same methodology were used to calculate the accuracy and precision with and without visual feedback, but without any subsequent motor training task.

### 2.6. Motor Training Tasks

A 3-day motor training task at home was initiated following session 1 (Figure 2) for twenty participants. The participants were asked to perform open-close jaw movements and finger lifting movements with the use of the customized plastic blocks (block 1 and block 2) to practice the target position with a metronome to maintain a constant speed. The exercise program for each day was to repeat each set of exercises for 10 times with the help of the plastic blocks with an interval of 2 minutes. Each participant was given a form with timetables to fill in with an “X” when the exercise was done for each day. After 3 days, the same jaw opening-closing and finger lifting movements were repeated two times (with and without visual feedback) in session 2 and assessed by the same examiner (Figure 2).

### 2.7. Statistical Analysis

The sample size was calculated a priori based on the detection of a minimum relevant difference of 25% at an *α* level of 0.05 and 80% power (i.e., the risk of a type I and type II error was 5% and 20%, respectively). A total of 20 participants were recruited in each group.

For each participant, the peak values of jaw and finger movements from each trial in the two sessions were determined by the K7 Program (Myotronics, Inc., USA).

The accuracy of the motor behaviors (*D*_accu_) was quantified by the ratio of the absolute error (*μ*) to the target value (*X*). It is expressed here as a percentage:

(1) $$\begin{array}{c} D_{\text{accu}}\left(\%\right)=\frac{\mu }{X}\times 100,\;\mu =\frac{1}{10}\overset{10}{\underset{i=1}{\sum }}\left|\left(X_{i}-X\right)\right|\;\left(i=1,2,3,\cdots ,9,10\right). \end{array}$$

The coefficient of variation (CV_prec_) was used to quantify the precision of the motor behaviors. The CV is the ratio of the standard deviation (*σ*) to their mean value ($\bar{x}$). It is expressed here as a percentage:

(2) $$\begin{array}{c} {\text{CV}}_{\text{prec}}\left(\%\right)=\frac{\sigma }{\bar{x}}\times 100,\;\bar{x}=\frac{1}{10}\overset{10}{\underset{i=1}{\sum }}x_{i},\;\sigma \sqrt{\frac{1}{10}\overset{10}{\underset{i=1}{\sum }}{\left(x_{i}-\bar{x}\right)}^{2}}\;\left(i=1,2,3,\cdots ,9,10\right).\\ \text{The relative changes of }D_{\text{accu}}\left(\%\right)=\frac{D_{\text{accu}‐\text{before}}-D_{\text{accu}‐\text{after}}}{D_{\text{accu}‐\text{before}}}\times 100.\\ \text{The relative changes of }{\text{CV}}_{\text{prec}}\left(\%\right)=\frac{{\text{CV}}_{\text{prec}‐\text{before}}‐\text{C}{\text{V}}_{\text{prec}‐\text{after}}}{{\text{CV}}_{\text{prec}‐\text{before}}}\times 100. \end{array}$$

The relative changes of *D*_accu_ and CV_prec_ in the two trials (with-without visual feedback) from session 1 to session 2 (before-after) were used to compare directly motor performance for jaw and finger movements.

Descriptive statistics were used to summarize the data. The necessary logarithmic transformation was performed when the data was not normally distributed. The mean and SDs of *D*_accu_, CV_prec_, and relative changes for jaw and finger movements in two trials (with visual feedback/without visual feedback) during session 1 and session 2 (before/after training) were calculated. A three-way analysis of variance (ANOVA) was used to analyze different outcome parameters with the following factors: session (2 levels: before and after training), site (2 levels: jaw and finger), and trial (2 levels: with and without visual feedback). A one-way ANOVA was used to evaluated differences for different amplitudes of the finger lifting task (4 levels: 10, 20, 40, and 60 mm). Post hoc tests were performed with an LSD Honest Significant Difference test with corrections for multiple comparisons. A value of *P* < 0.05 was considered statistically significant.

## 3. Results

**Figure 3:**
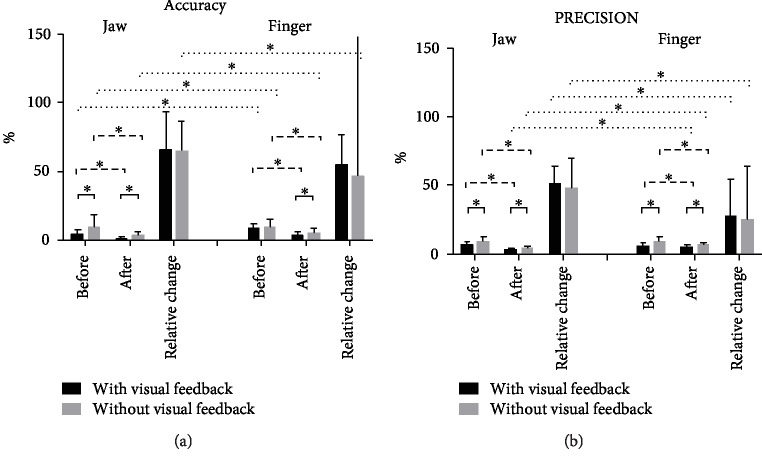
The mean and standard deviation of (a) *D*_accu_ (accuracy) and (b) CV_prec_ (precision) for different sessions (before, after, and relative changes) of different sites (jaw, finger) with or without visual feedback. ∗ indicates a significant difference (*P* < 0.05).

**Figure 4:**
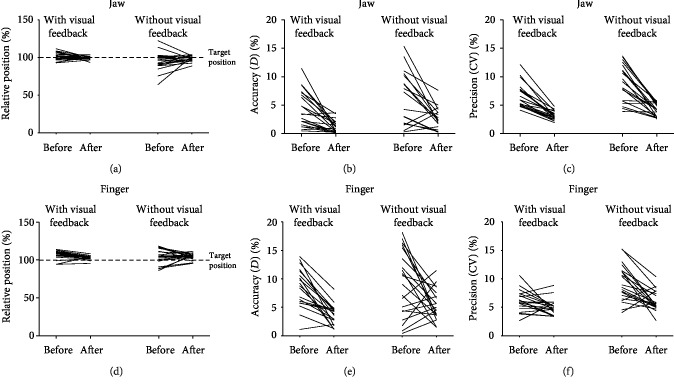
(a) Individual values of relative target position, (b) *D*_accu_ (accuracy), (c) CV_prec_ (precision) for jaw open-close movements and (d) individual values of relative target position, (e) *D*_accu_ (accuracy), and (f) CV_prec_ (precision) for finger lifting movements in percentage for 20 participants with or without visual feedback before and after 3-day motor training.

The mean and SDs of *D*_accu_, CV_prec_, and absolute changes (before-after) are shown in Table 1 and Figure 3. The mean and SDs of *D*_accu_ and CV_prec_ in 4 different amplitudes (10, 20, 40, and 60 mm) in two trials (with/without visual feedback) are shown in Table 2. All individual values for accuracy and precision are shown in Figure 4 and indicate improved performance; i.e., the individual target position values converge towards 100% for both jaw and finger movements, and *D*_accu_ and CV_prec_ decrease after the training tasks. The *D*_accu_ and CV_prec_ of jaw movement were 4.4 ± 3.2/6.8 ± 2.2 in trial 1 (with visual feedback) and 9.6 ± 9.2/9.2 ± 3.4 in trial 2 (without visual feedback) in session 1 (pretraining). In session 2 (posttraining), the *D*_accu_ and CV_prec_ were 1.2 ± 1.1/3.2 ± 0.8 in trial 1 and 3.1 ± 2.7/4.3 ± 1.1 in trial 2. The relative changes (before-after) were 65.6 ± 28.9/51.2 ± 12.5 in trial 1 and 65.1 ± 24.5/48.0 ± 22.1 in trial 2. *D*_accu_ of jaw and finger are significantly different at baseline (two types of movement pretraining with visual feedback). However, no significant differences of CV_prec_ are shown between the jaw and finger. So we compared the relative changes from pretraining to posttraining to show the differences in the motor control of the jaw and finger after the training period. The result shows that relative changes differ significantly from session 1 to session 2 between the jaw and finger. The value of the relative change of jaw movements was more than that of the finger showing that the control of the jaw is much better than that of the finger.

### 3.1. Comparison of Male and Female

Before motor training, we found that there were significant differences in the *D*_accu_ of jaw movements with visual feedback (ANOVA, *F* = 6.134, df = 1, *P* = 0.023) between males and females. However, males and females did not differ significantly in the CV_prec_ of jaw movement with visual feedback (ANOVA, *F* = 0.441, df = 1, *P* = 0.515) and the *D*_accu_ and CV_prec_ of jaw movements without visual feedback (ANOVA, *F* = 1.191, df = 1, *P* = 0.289; ANOVA, *F* = 1.145, df = 1, *P* = 0.299, respectively). There were no significant differences of the *D*_accu_ and CV_prec_ of finger movement with visual feedback (ANOVA, *F* = 0.611, df = 1, *P* = 0.444; ANOVA, *F* = 3.628, df = 1, *P* = 0.073, respectively) nor without visual feedback (ANOVA, *F* = 1.596, df = 1, *P* = 0.223; ANOVA, *F* = 0.114, df = 1, *P* = 0.739, respectively).

### 3.2. *D*_accu_

There were statistically significant effects of session (ANOVA, *F* = 41.183, df = 1, *P* < 0.001), trial (ANOVA, *F* = 11.795, df = 1, *P* = 0.001) and site (ANOVA, *F* = 8.736, df = 1, *P* = 0.004) for D_accu_.

In jaw open-close movements, D_accu_ in trial 2 (without visual feedback) was significantly higher than that in trial 1 (with visual feedback) in session 1 (pretraining) (ANOVA, *F* = 5.821, df = 1, *P* = 0.021) and session 2 (posttraining) (ANOVA, *F* = 8.150, df = 1, *P* = 0.007). Moreover, in trials 1 and 2, *D*_accu_ were higher in session 1 than in session 2 (ANOVA, *F* = 17.867, df = 1, *P* < 0.001; ANOVA, *F* = 9.316, df = 1, *P* = 0.004, respectively).

In finger lifting movements, *D*_accu_ in trial 2 was significantly higher than that in trial 1 in session 2 (ANOVA, *F* = 5.522, df = 1, *P* = 0.024) but not significantly different than that in session 1 (ANOVA, *F* = 0.337, df = 1, *P* = 0.565). *D*_accu_ in session 1 was significantly higher than that in session 2 both in trial 1 and in trial 2 (ANOVA, *F* = 28.613, df = 1, *P* < 0.001; ANOVA, *F* = 7.011, df = 1, *P* = 0.012, respectively).

The direct comparison of the finger lifting movements demonstrated significantly lower *D*_accu_ values than that of the jaw open-close movements (i.e., better accuracy) in trial 1 (ANOVA, *F* = 14.416, df = 1, *P* = 0.001) and trial 2 (ANOVA, *F* = 28.551, df = 1, *P* < 0.001) for session 1. *D*_accu_ for the jaw open-close movements was also significantly lower than that for the finger lifting movements in trial 2 (ANOVA, *F* = 7.511, df = 1, *P* = 0.009) for session 2 (Table 1). Moreover, the relative changes of *D*_accu_ from session 1 to session 2 (before-after training) in trial 2 were significantly different between jaw and finger movements (ANOVA; *F* = 6.211, df = 1, *P* = 0.017) indicating greater improvement in accuracy for the jaw movements compared to the finger movements from pre- to posttraining.

### 3.3. CV_prec_

For CV_prec_, there were also significant effects of session (ANOVA, *F* = 89.346, df = 1, *P* < 0.001), trial (ANOVA, *F* = 32.752, df = 1, *P* < 0.001), and site (ANOVA, *F* = 5.591, df = 1, *P* = 0.019). There were significant interactions between session and trial (ANOVA, *F* = 5.875, df = 1, *P* = 0.017), and the interaction between session and site was significant (ANOVA, *F* = 9.790, df = 1, *P* = 0.002) (Table 3).

CV_prec_ values for jaw open-close movements of trial 1 were lower than those of trial 2 in session 1 (ANOVA, *F* = 6.894, df = 1, *P* = 0.012) and session 2 (ANOVA, *F* = 13.114, df = 1, *P* = 0.001). In trials 1 and 2, CV_prec_ were lower in session 2 than in session 1 (ANOVA, *F* = 50.328, df = 1, *P* < 0.001; ANOVA, *F* = 38.546, df = 1, *P* < 0.001, respectively).

For finger lifting movements, CV_prec_ in trial 2 were significantly higher than those in trial 1 in session 1 (ANOVA, *F* = 14.572, df = 1, *P* < 0.001) and in session 2 (ANOVA, *F* = 5.166, df = 1, *P* = 0.029). CV_prec_ in session 1 were significantly higher than those in session 2 in trial 1 and trial 2 (ANOVA, *F* = 4.805, df = 1, *P* = 0.035; ANOVA, *F* = 14.479, df = 1, *P* = 0.001, respectively).

CV_prec_ values of jaw open-close movements were significantly lower in trial 1 (ANOVA, *F* = 26.302, df = 1, *P* < 0.001) and trial 2 (ANOVA, *F* = 16.283, df = 1, *P* < 0.001) for session 2 compared with those of finger lifting movements. There were no significant differences in trial 1 or trial 2 of session 2 (Table 1). The relative changes of CV_prec_ from session 1 to session 2 (before-after training) in trial 1 and trial 2 were both significantly different between jaw and finger movements (ANOVA, *F* = 12.670, df = 1, *P* = 0.001; ANOVA, *F* = 5.275, df = 1, *P* = 0.027, respectively) showing that improvements in precision for the jaw movements were more remarkable compared to those for the finger movements from pre- to posttraining.

### 3.4. Control Experiment

**Figure 5:**
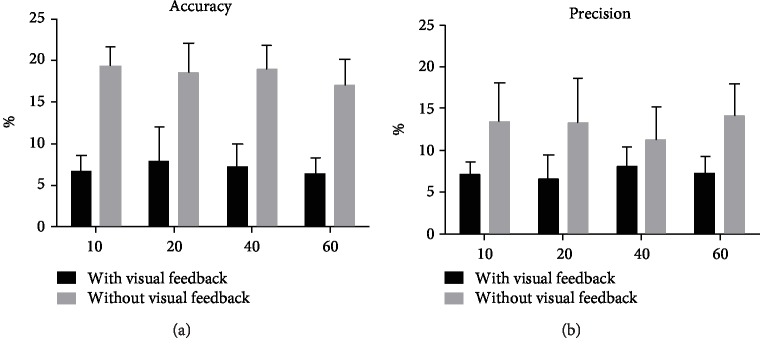
Control experiment (*n* = 11). The mean and standard deviation of (a) *D*_accu_ (accuracy) and (b) CV_prec_ (precision) for different amplitudes (10, 20, 40, and 60 mm) of finger movements with or without visual feedback.

The control experiment for the finger lifting movements at different amplitudes did not indicate any significant differences in *D*_accu_ or CV_prec_ neither with (ANOVA, df = 3, *F* = 0.552, *P* = 0.650; ANOVA, df = 3, *F* = 0.828, *P* = 0.486, respectively) nor without (ANOVA; df = 3, *F* = 1.232, *P* = 0.311; ANOVA, df = 3, *F* = 0.844, *P* = 0.478, respectively) visual feedback (Figure 5).

## 4. Discussion

The present study assessed jaw and finger motor performance during a short-term (3 days) task training and revealed a significant increase in the precision and accuracy of task performance for both the jaw and finger movements.

To promote learning of new abilities, retrieving lost skills or keeping existing skills is the purpose of neurorehabilitation but has received relatively little attention in the rehabilitation of impaired oral function, e.g., chewing and biting. A variety of factors may have influence on neurorehabilitation and effect on motor learning processes. These factors include, for example, verbal instructions and variability of training sessions, posture control, and visual feedback.

### 4.1. Neuroplasticity and Motor Improvement

The capacity of the nervous system to modify its organization to altered demands and environments has been termed “neuroplasticity” [21]. Neuroplasticity occurs when, for example, acquiring new skills, after damage to the nervous system and as a result of sensory deprivation. Neuroplasticity has been studied at different organizational levels of the nervous system, ranging from ion channels to synapses, neurons, neuronal columns, cortical maps, and behavior. These levels are, however, highly interlinked and interdependent. Associative learning, for example, induces changes in the release of neurotransmitters, which then may trigger a cascade of neurochemical events resulting in structural changes in the cerebral cortex such as the formation of new synapses or the reorganization of synaptic connections [22]. For instance, these structural changes could sometimes lead to an expansion of cortical maps [23]. Reorganizations of sensory cortical maps have been linked to changes in perceptual abilities measured at the behavioral level [24]. Neuroplasticity is a continuous process allowing short-term, medium-term, and long-term remodeling of the neuron-synaptic organization, with the aim of optimizing the function of neural networks during phylogenesis, ontogeny, and physiologic learning and following brain injury. Previous research has demonstrated that primary motor area (MI) neuroplasticity, as reflected in changes in intracortical microstimulation (ICMS) features or neuronal activity patterns, is associated with the learning of a novel orofacial motor task. Cortical damage, stroke, can impair orofacial sensory and motor functions in humans, which may substantiate that MI plays a strategic role not only in elemental and learned motor behaviors but also in certain aspects of chewing and swallowing, a kind of peripheral region change. The studies have utilized ICMS, reversible cold block, or single neuron recordings in MI. MI is contributing to control and regulation of mastication and swallowing which may help to explain why cortical damage, stroke, can impair orofacial sensory and motor functions in humans [25].

Motor improvement also reflects other functional changes not necessarily neuroplastic ones. Previous research in rats demonstrated that increases in forelimb motor activity can induce cortical angiogenesis which suggest that changes occur not only in neurons but also occur in glial cells and blood vessels [26, 27]. Besides, motor control training may improve the size and function of trunk muscles in elite football players which is a kind of peripheral changes [28]. The present data may however not easily be explained by such peripheral changes, and more likely, central neuroplasticity plays a more important role in the behavioral improvements observed for both the jaw and finger movements.

### 4.2. Effects of Visual Feedback

It is unclear whether cognition and motor control are parallel and interactive or serial and independent processes. According to one view, cognitive control refers to a set of modality-nonspecific processes that act on supramodal representations and precede response modality-specific motor processes [29]. In the present study, it was shown that observation of your own jaw and finger movements to reach a specific target position was associated with more accurate and precise movements compared to when no visual cues were provided. Interestingly, this was observed for both the jaw and finger movements; however, in more daily activities, visual inspection of your fingers is obviously feasible whereas a mirror is required to allow a visual inspection of your jaw movements. Nevertheless, both the spinal and trigeminal systems seem to perform better when under visual control. In terms of development of specific training paradigms for neurorehabilitation of lost jaw function, it seems valuable to use mirrors for individuals to obtain visual feedback.

Participants can, indeed, acquire neural representations on the basis of visual information [30]. Further, while motor learning by observation does not depend on conscious awareness of the individuals, the tendency for an unrelated movement task to significantly reduce the ability of participants to learn by observing indicates that the implicit engagement of motor systems is required [17].

It has been demonstrated that the force control of jaw muscles could be improved when the isometric contractions of muscles were guided with visual feedback [31, 32]. In a previous study, there were indications that observation of a movement activates cortical motor areas involved in producing the same movement [33]. This suggests that motor systems are involved in acquiring neural representations during observation, which also will have occurred in the present study for both the jaw- and finger-movement tasks.

### 4.3. Effects of Training

Motor adaptation of the jaw motor system has been studied by many investigators [34, 35]. It has been suggested that it would be used in the future with a great development prospect to get adaptation during a long-term training by undergoing force-controlled balancing tasks [35]. In the study reported by Kumar et al. [14], increased precision and shorter duration of the tasks were observed following a short-term training task (about 30 min), which may suggest a better fine oral motor control and optimization of jaw movements. A study using transcranial magnetic stimulation (TMS) to probe cortical excitability demonstrated that repeated and standardized tooth-clenching tasks triggered significant neuroplastic changes in the corticomotor control of the jaw-closing muscles [5].

In the present study, significant differences were found for both jaw and finger movements between pre- and posttraining. Participants executed the repeated movements more accurately and precisely after they underwent a 3-day motor training program. It has been demonstrated that motor learning is highly related to motor cortex plasticity [36], and a previous study suggested that neuroplastic change occurs in the corticomotor pathways within 30 min after a repetitive tongue training task [4].

It seems reasonable to suggest that practice of repeated movements or tasks is essential for learning and acquisition of new motor skills [37]. Indeed, skilled motor training will include the optimization of different action phases [38] and also lead to cortical reorganization and adaptation of the behavior of single motor units [8, 39]. Acquisition of skilled movement normally refers to processes by which movements performed through repeated practice will be executed more effortlessly and it may also be associated with significant reorganization of the motor cortex [40]. In this study, only vertical jaw and finger movements were recorded, and further studies will be needed to directly demonstrate any neuroplastic changes underlying the improved performance for both the jaw and finger, e.g., using TMS or fMRI studies.

### 4.4. Differences between Jaw and Finger Movements

It was observed that both the *D*_accu_ (accuracy) and CV_prec_ values (precision) were significantly different between jaw and finger movements. Indeed, the differences for the changes of *D*_accu_ and CV_prec_ were significantly different from session 1 to session 2 between the jaw and finger and demonstrated that the accuracy and precision for jaw open-close movements were improved more than those for finger lifting movements after participants had trained for 3 days. There are several distinctive properties of the jaw muscles and their control that distinguish them from the spinally innervated neck, trunk, and limb muscles. In terms of movement and sensorimotor control, orofacial muscles contract bilaterally and are under bilateral sensorimotor control; limb muscles contract unilaterally or alternating left/right contraction and are under unilateral sensorimotor control. According to the location of the motor neuron cell bodies, orofacial muscles are controlled from distinctive brainstem motor nuclei, and limb muscles are controlled from the ventral horn of the spinal cord. Besides, with regard to muscle spindles and some periodontal mechanoreceptors, orofacial muscles are controlled from neurons within the CNS (in the mesencephalic nucleus), and limb muscles are controlled outside the CNS [41]. Previous studies also showed that a distinct difference was observed in the mechanisms of motor control in the trigeminally innervated jaw muscles compared to those in spinally innervated finger muscles when similar motor tasks were performed [15, 42]. It has been suggested that this may lead to differences in motor patterns and control mechanisms between spinally innervated and trigeminally innervated muscle contractions. In the study comparing the cortical activity of bilateral light fist clenching and light tooth clenching in healthy individuals [16], there were indications that the tooth clenching task was more complex than the hand motor task in terms of the associated cortical activation patterns [16]. It was suggested that the jaw motor system may be a more complex system than the finger. The present findings may be interpreted in the same manner namely that improvements of performance are more likely to occur in the jaw motor system after short-term training than the hand motor system.

However, the recent findings from a study of Kumar et al. [15] reported that force control within the jaw motor system was more variable than the fingers indicating disassociation between accuracy and precision due to repeated performance of an oral motor task and digital motor task. It was proposed that there may be mechanistic differences between force and motor control as such.

### 4.5. Methodological Limitations

Many factors may have effects on motor learning including age, anatomical characteristics, specifics of the training sessions, the individual's active participation and motivation, positive and negative learning transfer, posture control, and memory. An element of a test-retest measurement incorporated in the outcome needs to be considered for this kind of training studies. All these factors may be clinically relevant and may provide the basis for emerging or new lines of research having to do with retraining of sensorimotor function in neurological patients and in patients undergoing oral rehabilitation [25, 43]. In this study, only one direction of movement was trained and tested, and the sample size was relatively small with only young and healthy participants being included. Another limitation in the present study could be the fixed amplitude (20 mm) for the finger movement when the amplitude for the jaw movement was set to 50% of the individual maximal jaw opening. Although no differences in accuracy and precision were found between different ranges of finger lift movements, including 50% of the maximal finger lift capacity in the control experiment in this study, one could argue that the jaw and finger movements were still quite different making a direct comparison difficult. However, in terms of the absolute jaw opening distance, 50% corresponds approximately to 20-25 mm [44–46]. In combination with the fact that accuracy and precision did not change over a range of different finger lift movements, it seems that the present findings are valid and, indeed, suggest a remarkable difference in training-evoked effects between the spinal and trigeminal motor systems.

Another issue to be discussed in this study is related to the participant's memory of the different movements. According to Ebbinghaus' classical forgetting curve, memory retention reduced from 100% to 58.2% only after 20 min after the first learning task [47]. Memory mechanisms may also play an important role in motor learning and training. However, in the present study, all series of movements were performed in a few minutes after the initial learning so that further studies would be needed to demonstrate a clear decreasing or increasing trend in performance in the consecutive movements at different time points. It should be mentioned that the outcome parameters in the present study also may include the natural variability of the task performance; however, this is inherent in the study of training-related effects due to the repeated task performance. In addition, the fingers work very often under a visual control, while the jaw works almost always without visual feedback. The influence of visual feedback was different between spinally innervated and trigeminally innervated muscles, and the specific mechanism of force execution from muscle activity seems to be different between the jaw and hand motor systems, which may affect the results of the experiment; so further follow-up research could explore potential mechanisms related to the force control of hand and jaw muscles in the peripheral nervous system [17]. Finally, further studies would also be needed to examine the effect of motor cortex neuroplasticity related to longer training paradigms and longer follow-up periods and in larger sample sizes and with more complex types of movement training.

## 5. Conclusion

In conclusion, the accuracy and precision of standardized jaw and finger movements are dependent on visual feedback and appear to improve more by training in the trigeminal system. Somatosensory plus visual feedback, in addition to proprioception, provides a better sense of body (jaw and finger) position before a movement is being carried-out; sensory inputs and subsequent sensorimotor integration allow for corrections in movements while they occur; if repeated enough times, at enough intensity, these may generate plastic changes, such as generation of new networks, reorganization of motor representations, and changes in neuron excitability which will lead to changes in motor programs so that future movements are performed more accurately and precisely. Each of the above changes in motor functions involves different plastic changes in the brain occurring at different points of time.

## Figures and Tables

**Table 1:** Mean values and standard deviation of *D*_accu_ and CV_prec_ and relative changes in *D*_accu_ and CV_prec_ of 20 participants for jaw open-close and finger lifting movements with or without visual feedback before or after 3-day motor training.

Site	Session (training)	Trial (visual feedback)	*D*_accu_ (%)	CV_prec_ (%)
Jaw	Before	With	4.4 ± 3.2^abc^	6.8 ± 2.2^ab^
		Without	9.6 ± 9.2^bc^	9.2 ± 3.4^b^
	After	With	1.2 ± 1.1^a^	3.2 ± 0.8^ac^
		Without	3.1 ± 2.7^c^	4.3 ± 1.1^c^
	Relative change	With	65.6 ± 28.9	51.2 ± 12.5^c^
		Without	65.1 ± 24.5^c^	48.0 ± 22.1^c^

Finger	Before	With	8.3 ± 3.4^b^	6.1 ± 1.9^ab^
		Without	9.2 ± 5.8^b^	9.3 ± 3.2^b^
	After	With	3.7 ± 1.8^a^	5.0 ± 1.4^a^
		Without	5.4 ± 2.7	6.2 ± 1.8
	Relative change	With	54.4 ± 22.4	27.8 ± 26.6
		Without	46.3 ± 198.4	25.0 ± 39.1

Letters refer to significant differences between trials (a), sessions (b), and sites (c). *D*_accu_: accuracy; CV_prec_: precision.

**Table 2:** Comparisons of *D*_accu_ and CV_prec_ in 4 in different amplitudes (10, 20, 40, and 60 mm) in two trials (with/without visual feedback).

Length	Trial (visual feedback)	*D*_accu_	CV_prec_
10	With	6.62 ± 1.86	7.10 ± 1.47
	Without	19.31 ± 2.3	13.4 ± 4.43

20	With	7.84 ± 4.01	6.51 ± 2.81
	Without	18.54 ± 3.41	13.23 ± 5.16

40	With	7.26 ± 2.53	8.04 ± 2.27
	Without	18.89 ± 2.76	11.16 ± 3.78

60	With	6.45 ± 1.78	7.22 ± 2
	Without	16.97 ± 3.04	14.05 ± 3.7

**Table 3:** Comparisons of *D*_accu_ and CV_prec_ in 20 participants at two sites (jaw and finger) in two trials (with/without visual feedback) over two sessions (before/after 3-day training) by three-way factorial analysis of variance (ANOVA).

	*D*_accu_		CV_prec_	
	*F*	*P*	*F*	*P*
Session	41.183	<0.001^∗^	89.346	<0.001^∗^
Trial	11.795	0.001^∗^	32.752	<0.001^∗^
Site	8.736	0.004^∗^	5.591	0.019^∗^
Session∗trial	0.836	0.362	5.875	0.017^∗^
Session∗site	0.218	0.641	9.790	0.002^∗^
Trial∗site	2.586	0.110	0.460	0.499
Session∗trial∗site	2.259	0.135	0.313	0.577

∗ indicates a significant difference (*P* < 0.05).

## Data Availability

Raw data were generated using a kinesiograph (K7/CMS; Myotronics, Inc., USA) and commercial software program (K7 Program, Myotronics, Inc., USA). Derived data supporting the findings of this study are available from the corresponding authors upon request.
