# Corrigendum to “Effects of Motor Training on Accuracy and Precision of Jaw and Finger Movements”

**DOI:** 10.1155/2021/9760392

**Published:** 2021-07-24

**Authors:** Yinan Chen, Song Wu, Zhengting Tang, Jinglu Zhang, Lin Wang, Linfeng Yu, Kelun Wang, Peter Svensson

**Affiliations:** ^1^Orofacial Pain & TMD Research Unit, Institute of Stomatology, Department of Polyclinic, Affiliated Hospital of Stomatology, Nanjing Medical University, Nanjing, China; ^2^Jiangsu Key Laboratory of Oral Diseases, Nanjing Medical University, Department of Polyclinic, Affiliated Hospital of Stomatology, Nanjing Medical University, Nanjing, China; ^3^Department of Dentistry, BenQ Medical Center, The Affiliated BenQ Hospital of Nanjing Medical University, Nanjing, Jiangsu Province, China; ^4^Center for Sensory-Motor Interaction (SMI), Aalborg University, Aalborg, Denmark; ^5^Section of Orofacial Pain and Jaw Function, Department of Dentistry and Oral Health, Aarhus University, Denmark; ^6^Department of Dental Medicine, Karolinska Institutet, Huddinge, Sweden; ^7^Scandinavian Center for Orofacial Neurosciences (SCON), Denmark

In the article titled “Effects of Motor Training on Accuracy and Precision of Jaw and Finger Movements” [1], Dr. Jinglu Zhang was incorrectly listed as the corresponding author. The corresponding author is Dr. Lin Wang, as shown above.
